# Embodied decisions unfolding over time: a meta-ethnography systematic review of people with cancer’s reasons for delaying or declining end-of-life care

**DOI:** 10.1186/s12904-024-01342-5

**Published:** 2024-02-19

**Authors:** Jessica Young, Antonia Lyons, Richard Egan, Kevin Dew

**Affiliations:** 1 Victoria University of Wellington, Wellington, New Zealand; 2 University of Auckland, Auckland, New Zealand; 3 University of Otago, Dunedin, New Zealand

**Keywords:** Systematic review, Meta-ethnography, Hospice, Decision-making, Cancer, Palliative care, End-of-life, Patient experience

## Abstract

**Background:**

Barriers to accessing hospice and palliative care have been well studied. An important yet less researched area is why people approaching the end-of-life decline a referral when they are offered services. This review focused on synthesising literature on patients in the last months of life due to a cancer diagnosis who have declined a referral to end-of-life care.

**Methods:**

Six academic databases were systematically searched for qualitative literature published between 2007 and 2021. Two researchers independently reviewed and critically appraised the studies. Using meta-ethnographic methods of translation and synthesis, we set out to identify and develop a new overarching model of the reasons patients decline end-of-life care and the factors contributing to this decision.

**Results:**

The search yielded 2060 articles, and nine articles were identified that met the review inclusion criteria. The included studies can be reconceptualised with the key concept of ‘embodied decisions unfolding over time’. It emphasises the iterative, dynamic, situational, contextual and relational nature of decisions about end-of-life care that are grounded in people’s physical experiences. The primary influences on how that decision unfolded for patients were (1) the communication they received about end-of-life care; (2) uncertainty around their prognosis, and (3) the evolving situations in which the patient and family found themselves. Our review identified contextual, person and medical factors that helped to shape the decision-making process.

**Conclusions:**

Decisions about when (and for some, whether at all) to accept end-of-life care are made in a complex system with preferences shifting over time, in relation to the embodied experience of life-limiting cancer. Time is central to patients’ end-of-life care decision-making, in particular estimating how much time one has left and patients’ embodied knowing about when the right time for end-of-life care is. The multiple and intersecting domains of health that inform decision-making, namely physical, mental, social, and existential/spiritual as well as emotions/affect need further exploration. The integration of palliative care across the cancer care trajectory and earlier introduction of end-of-life care highlight the importance of these findings for improving access whilst recognising that accessing end-of-life care will not be desired by all patients.

## Introduction

People with life-limiting cancer have some of the greatest need for symptom management [1] yet many do not accept end-of-life care. For example in the United States (US), as many of 30% of eligible patients decline hospice care [2]. End-of-life care decision-making is significant because of the intersecting service provision; one study in New Zealand found that people who received support from hospice were more likely to receive support from multiple other services [3]. 

The terms hospice and palliative care are often used interchangeably or combined to hospice palliative care [4]. While hospice care can be delivered at home or in a hospice facility, it tends to focus on care in the last months of life [5]. In contrast, palliative care is now considered best practice for all people with serious illness early in the disease course and is usually not limited to the last months of life [1, 6]. According to Hawley, writing from the Canadian context and reflecting on North America, a consistent feature differentiating hospice from palliative care is that hospice is for patients who are at end of life and have ceased curative treatments; palliative care is for patients who are not yet at end of life [7]. However this is not the case in all countries, for example in the United Kingdom (UK) where palliative care is available following a life-limiting diagnosis, it is typically discussed with a greater emphasis towards the end of life [8]. Recognising the debates of terminology and the varying definitions of hospice and palliative care around the world, for the purposes of this review we are using end-of-life care to refer to specialist care provided anywhere for people in the last months of life [5]. 

A number of systematic reviews have been undertaken to examine the barriers of accessing end-of-life care and the difficulty of the transition away from curative care [7, 9, 10]. This work consistently demonstrates a number of barriers to access, including lack of awareness or knowledge of the service, reluctance from referrers, family and patients, timeliness of referrals particularly for minority and structurally disadvantaged populations, eligibility criteria, resource availability, cultural appropriateness of the services and unclear dying trajectories [2, 7, 9, 10]. Previous work has reviewed research published during 1997–2003 and 2004–2012 thus an updated review examining barriers to end-of-life care is timely [9, 10]. However, an important yet less researched area is why people decline a referral when they are offered services. Therefore, the current review focused on people’s experiences of declining end-of-life care. It employed meta-ethnography as the review methodology, enabling it to capture in-depth accounts and experiences [11–13]. 

The aim of this systematic review is to review and synthesise the literature published over the past 15 years regarding what is known about cancer patients’ reasons for declining end-of-life care. Using meta-ethnographic methods of translation and synthesis, we set out to identify and develop a new overarching model of the reasons patients decline end-of-life care and the factors contributing to this decision. The guiding questions for the review were:

1. Why do patients with life-limiting cancer decline end-of-life care ?
2. What informed their decision to decline end-of-life care?
3. What are their experiences of making the decision to decline end-of-life care?
4. Are there common characteristics of this population?

## Method

This review focused on patients approaching the end of life due to a cancer diagnosis who have declined a referral to end-of-life care. We registered the review on PROSPERO (CRD42022310809).

### Databases

With the assistance of a subject librarian, we searched the following electronic bibliographic databases: Web of Science, Scopus, ProQuest, Embase (OVID), Medline (OVID), CINAHL.

### Search terms

Palliative care OR hospice OR end-of-life care; AND decision-making; AND cancer; AND patients; AND experiences. The searches were run in November 2021 and citations again before submission (October 2022). Although we were not focussing on palliative care as defined above, it was included as a search term to ensure we captured any potential articles that may have been eligible because as noted, the terms hospice and palliative care are often used interchangeably in the published literature and may be referring to the type of end-of-life care we were focusing on.

### Parameters

The inclusion and exclusion criteria are shown in Table 1.

**Table 1:** Inclusion and exclusion criteria

Inclusion	Exclusion
English language	Primarily non-cancer patient sample
Published 2007–2021	Only focussing on caregivers or health care professionals’ experiences
Patients with life-limiting cancer	Inadequate reporting on end-of-life care decision-making
Qualitative studies, mixed methods	Randomised-controlled trials, non-randomised trials, cohort studies (prospective and retrospective), editorials, letters to the editor, comments, narrative case reports, conferences, and lectures

These dates were chosen because no reviews on the reasons for declining end-of-life care have been published covering this time period. Further, earlier reviews focused on barriers to end-of-life care enrolment, not exclusively on people declining a referral when they are offered services. Reference lists and citations of included studies using Google Scholar were hand-searched, as were our own personal archives. For end-of-life care and non-end-of-life care comparison studies, we extracted pertinent data if it helped to answer the research questions. Reviews were excluded but read and checked for any references that our searches missed.

### Participants/population

To keep the review manageable, we chose to focus on patients with life-limiting cancer because much of the research focuses on this population. This group tend to be well-served by health and support services [3]. 

### Data extraction (selection and coding)

#### Data management

Rayyan, a systematic review management software programme, was used for de-duplication, inclusion/exclusion management at the title and abstract stage, and for collaboration between the research team.

#### Selection process

Two study team [JY, AL] members independently reviewed titles and abstracts to identify studies that may meet inclusion criteria. Full texts were reviewed to confirm eligibility. Discrepancies were to be reviewed by a third study team member, though this was not necessary.

#### Data collection

Study characteristics and first order content and second order constructs were extracted and collated for further synthesis (see Tables 2 and 3).

**Table 2:** Summary of the included studies

Authors Year Location	Aim/RQ	Eligible study participants and data collection methods	Data analysis methods	Key findings (second order constructs)
Vig et al. 2010 US [14]	To identify reasons that eligible patients do not enroll in hospice	Semi-structured interviews 10 non-end-of-life care patients	Content analysis	**Patient/Family Perceptions** • Patient and/or family “not ready” for hospice • Misconception that hospice care is for the last hours to days of life • Hospice means acknowledging dying • Waiting to hear about any other treatment options from doctor(s) • Spouse wants help from hospice, patient doesn’t • Wives protective of their caregiving role • Family concerns about their ability to care for patient at home • Family not sure what hospice could add to existing care **Hospice Specific** • Definition of the hospice appropriate patient • Requiring patients/families to choose between hospice or palliative treatment • How hospice is presented during the initial visit • Hospice referral confused with a home health referral • Hospice informational visit confused with a hospice admissions visit **Systems Issues** • Patient concerns about continuity of care after hospice enrollment • Inadequate hospice benefit from private insurance • Delay in obtaining physician order for hospice
Carrion 2010 US [15]	The extent to which individuals and families in the Latino community are involved with institutionalized assistance from hospice, and the barriers to referral which can be attributed to organizational structure, language, and culture	Semi-structured interviews 10 non-end-of-life care patients	Open coding and thematic categorization	**Structural organizational barriers**: health literacy, invasion of privacy, and response time. **Factors that impeded Latino families from utilizing of hospice services**: place of referral-office, cultural beliefs, and paid caregivers. **Language**: Need for Spanish-speaking staff (by both hospice and non-hospice users) and educational hospice materials in Spanish.
Chapple et al. 2011 UK [16]	To explore why some of these patients reported a preference for end of life care and death either at home, hospice, nursing home or hospital, how strongly they felt about this, and how they described making decisions.	Semi-structured interviews 8 people with life-limiting pancreatic cancer	Thematic analysis	Care available at home; experience of hospital care; perceptions and experiences of hospice care; fears of negative associations with place of death (for family)
Frey et al. 2013 NZ [17]	The aim of the study is to identify challenges to the use of hospice services for Maori, Pacific and Asian patients.	Semi-structured interviews 7 non-end-of-life care patients	Thematic analysis	**A matter of culture**: awareness of hospice services; access to information; misinformation; cultural understandings.
Meeker et al. 2014 US [18]	The purpose of this study was to explore patients’ and caregivers’ experiences and perspectives as they responded to advanced illness and, when relevant, transitioned to comfort-focused care.	Semi-structured interviews with mostly dyads, some patient only 12 patients (10 with cancer) that included hospitalized adult patients who met the end-of-life care eligibility criteria but who had not chosen to focus on comfort care or elected end-of-life care or had only done so within the past week.	Constant comparative techniques of grounded theory	**Recursive process of contending with advanced illness**: Suffering (shared forms of suffering, reciprocal suffering), struggling (enduring, fighting), and either continuing to struggle and suffer or some moved into the settling phase (adjusting, awareness of terminality)
Waldrop et al. 2015 US [19]	The purpose of this study was to compare decision-making in late-stage cancer in people who enrolled in hospice with those who declined. Concepts from the Carroll and Johnson (1990) decision-making framework guided the development of a hospice decision-making model.	Qualitative and quantatitive - interviews (open ended questions and scaled measures) 24 non-end-of-life carepatients	theory led thematic analysis using the Carroll and Johnson (1990) model and constant comparative analysis.	The adapted Carroll and Johnson (1990) model presents the stages of: Recognition of Advanced Cancer and Information and Communication as ongoing are experienced similarly by both hospice and non-hospice groups. There was recursive relationship between the stages Formulation of Awareness and Generation of Alternatives that informed the Evaluation of Hospice (in the future, I will know when it is time); these stages were different in the hospice and non-hospice groups
Lin et al. 2019 Taiwan [20]	The aim of this study was therefore to explore the decision-making processes and drivers associated with receiving palliative care in advance care planning discussions from perspectives of people living with advanced cancer, their families and healthcare professionals in northern Taiwan.	Semi-structured interviews 15 patients with advanced cancer in oncology or hospice unit	Thematic analysis	The decisions for not choosing palliative care as part of advance care planning discussions are driven by: patients weighing others’ benefits more important than benefits to themselves; trying to pacify the families; being a role model for children by facing the challenge of active treatment.
Spencer et al. 2020 US [21]	To further understandings of how hospice decisions unfold over time, consistent with recent calls for studying the complexity involved in end-of-life care.	Semi-structured interviews 20 non-end-of-life care patients	Abductive framework analysis with a phenomenological perspective	Propose the term a “soft no,” in which patients neither accept nor overtly refuse hospice. Those giving “soft” refusals do not explicitly refuse hospice, but their actions function to postpone a hospice decision in an uncertain health context that may become more clear over time. (1) not seeing the value added of hospice (yet), (2) assuming the timing is premature (not dying yet), and (3) relying on extensive health-related support networks that justify or endorse continuation of active care.
Pini et al. 2021 [22] UK	The aim of this paper was to identify current barriers, facilitators and experiences of raising and discussing palliative care with people with advanced cancer.	Semi-structured interviews, non-referred had own interview schedule 8 non-end-of-life care patients	Framework analysis	**Referral process**: timing and triggers; responsibility. **Engagement**: perception of treatment, prognosis and Palliative Care; psychological and emotional preparedness for discussion; understanding how palliative care could benefit present and future.

NB. We have retained the original terminology of the studies in the table

**Table 3:** Themes identified in included studies

Themes/constructs	Reviewed papers (in publication order)								
	Vig et al. (2010) [14]	Carrion (2010) [15]	Chapple et al. (2011) [16]	Frey et al. (2013) [17]	Meeker et al. (2014) [18]	Waldrop et al. (2015) [19]	Lin et al. (2019) [20]	Pini et al. (2020) [22]	Spencer et al. (2020) [21]
**Influences**									
**Person Factors**									
identity		✔		✔	✔	✔			
hope		✔			✔	✔	✔	✔	
emotions	✔	✔	✔	✔	✔	✔	✔	✔	
preferences	✔	✔	✔	✔	✔			✔	✔
quality of life / suffering	✔		✔		✔	✔	✔	✔	✔
readiness / preparedness	✔	✔				✔	✔	✔	✔
end-of-life care knowledge & perceptions	✔	✔	✔	✔			✔	✔	✔
**Medical factors**									
treatment options	✔	✔	✔	✔	✔	✔	✔	✔	✔
prognosis	✔	✔			✔	✔	✔	✔	
relationships with providers	✔		✔	✔				✔	✔
trust	✔		✔	✔	✔		✔		✔
**Contextual factors**	✔	✔	✔	✔	✔	✔	✔		✔
**Interpretive themes**									
** Communication: timing, services and content**	✔	✔		✔	✔	✔	✔	✔	✔
** Uncertainty**						✔	✔	✔	✔
** Evolving situations**			✔		✔	✔			✔
**Key construct**									
** Decisions unfolding over time**					✔	✔			✔

Bold text indicates third order interpretive themes and key concepts developed by the review authors and non-bold text indicates second order constructs from the included articles

### Quality assessment

A Critical Appraisal Skills Programme (CASP) quality assessment for qualitative studies checklist was applied to all studies to assess the quality. The CASP guidelines provides detailed instructions on how to interpret the criteria to assess the rigour, credibility and relevance of each study. All were found to be of sufficient quality (see Table 4).

**Table 4:** CASP quality assessment for included studies

	Vig et al. (2010) [14]	Carrion (2010) [15]	Chapple et al. (2011) [16]	Frey et al. (2013) [17]	Meeker et al. (2014) [18]	Waldrop et al. (2015)^a^ [19]	Lin et al. (2019) [20]	Pini et al. (2020) [22]	Spencer et al. (2020) [21]
**Section A: Are the results valid?**									
1. Was there a clear statement of the aims of the research?	Yes	Yes	Yes	Yes	Yes	Yes	Yes	Yes	Yes
2. Is a qualitative methodology appropriate?	Yes	Yes	Yes	Yes	Yes	Yes	Yes	Yes	Yes
**Is it worth continuing?**									
3. Was the research design appropriate to address the aims of the research?	Yes	Yes	Yes	Yes	Yes	Yes	Yes	Yes	Yes
4. Was the recruitment strategy appropriate to the aims of the research?	Yes	Yes	Yes	Yes	Yes	Yes	Yes	Yes	Yes
5. Was the data collected in a way that addressed the research issue?	Yes	Yes	Yes	Yes	Yes	Yes	Yes	Yes	Yes
6. Has the relationship between researcher and participants been adequately considered?	Yes	Yes	Not described	Yes	Yes	Yes	Yes	Not described	Yes
**Section B: What are the results?**									
7. Have the ethical issues been taken into consideration?	Yes	Yes	Yes	Yes	Yes	Yes	Yes	Yes	Yes
8. Was the data analysis sufficiently rigorous?	Yes	Yes	Yes	Yes	Yes	Yes	Yes	Yes	Yes
9. Is there a clear statement of findings?	Yes	Yes	Yes	Yes	Yes	Yes	Yes	Yes	Yes
**Section C: Will the results help locally?**									
10. How valuable is the research?^b^	✔✔	✔✔	✔	✔✔	✔✔	✔✔	✔✔	✔✔	✔✔

^a^Only evaluating the qualitative component of the research which they described as "dominant"

^b^Two ticks indicates very valuable, one tick indicates partially valuable

### Analysis

We selected a meta-ethnography analysis because it was the most suitable approach to answer the questions posed by this review. No other previous systematic reviews in this area have used this approach [9, 10]. Rather than summarising the literature, a meta-ethnography aggregates and inductively re-interprets the data, concepts and themes. This methodology is most useful for developing conceptual models and theories. The goal of producing a higher order interpretation is to make a valuable conceptual contribution to the literature that is grounded in existing evidence [11–13]. Meta-ethnography has been used successfully to produce new interpretations of existing studies and advance current understandings of a phenomenon, including in other areas of end-of-life care [23, 24]. 

We used the metaethnography phases first developed by Noblit and Hare, and elaborated upon by Sattar et al. and Atkins et al. [11–13] Early phases in this meta-ethnographic approach involved extracting the first order (verbatim participant quotations) and second-order constructs (themes interpreted by authors) from each primary qualitative study into an excel spreadsheet along with study characteristics (see Tables 2 and 3). We arranged the articles chronologically and created a list of themes to examine common and recurring concepts across the included articles, using a constant comparison approach to identify new themes.

The fourth phase looked across the studies to translate them into each other to understand how they are related. This involved comparing the themes from paper one (in date order, with the earliest first) with paper two, how paper three fitted with the combination of papers one and two and so on. Here we followed Atkins et al.’s method to reduce the themes into categories as the analysis progressed and kept an open mind for identifying emerging themes [12]. We reduced the themes into categories as the analysis progressed and inductively started to identify categories that may become the third order constructs.

The fifth phase involved conducting reciprocal, refutational and line of synthesis translations. “Reciprocal translation occurs when concepts in one study can *incorporate* those of another, whereas a refutational translation explains and explores differences, exceptions, incongruities and *inconsistencies*” [emphasis added] [12]. We discussed, annotated and systematically compared each article’s themes, supplemented by relevant first order data. From these processes of comparing differences and similarities as well as the gaps we noted, we produced a model of the key categories and themes, combining the third-order constructs (interpretations developed by reviewers from a tertiary analysis of the first order content and second order constructs) in a line of synthesis of translation, as shown below [11]. The synthesis of themes into a model makes the whole more than the sum of its parts alone.

## Results

**Fig. 1:**
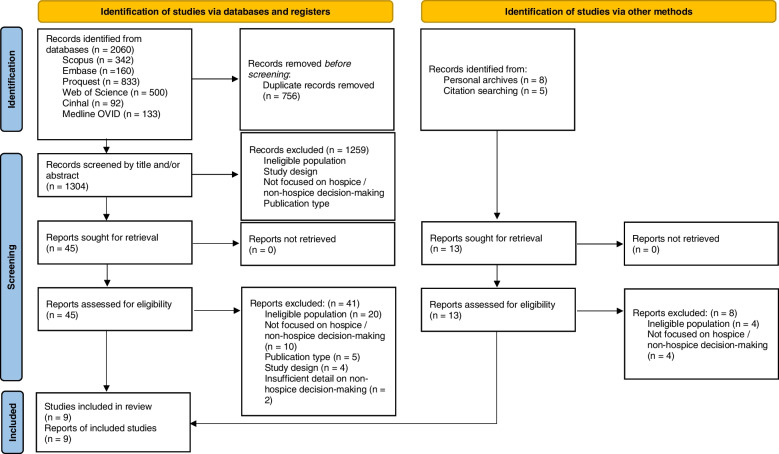
PRISMA 2020 flow diagram

The search results yielded 2060 articles. Following the application of the inclusion and exclusion criteriafour articles were eligible for inclusion. Our personal reference software databases were also searched and identified a further four articles for inclusion. Hand searches of reference lists and citations of the selected articles produced one additional study for inclusion (see Fig. 1). No new shared keywords were identified from the additional articles that enhanced the search. Key words on the additional articles used much broader MeSH terms that included subterms previously used in the search (e.g. terminal care is a borader MeSH term that covers wide-ranging concepts and subterms, including hospice) or non-MeSH terms (e.g. end-of-life transitions). Our review therefore included nine articles that reported independent studies involving cancer patients who had declined end-of-life care.

Studies took place in the United States [14, 15, 18, 19, 21], UK [16, 22], New Zealand [17] and Taiwan [20]. Participants were recruited to the studies through oncology and palliative care hospital settings, palliative care outpatient clinics, general practice as well as community workers and groups, newsletters, and notice boards. No studies focused on aged residential care. One study was ‘qualitative-dominant’ mixed methods using interviews and the Katz, QLQ-30 and Lubben Social Network scales to measure functional ability, quality of life and social support respectively in combination with the Carroll & Johnson decision-making model [19]. The remaining studies all employed semi-structured interviews. Analysis of qualitative data varied, and included content analysis, framework analysis, abductive framework analysis with a phenomenological perspective, thematic analysis, theory-led thematic analysis, and constant comparison.

All studies included patients and some also included family members [16, 18, 19, 21]. We focused on the patient perspectives and supplemented these with family perspectives where no patient perspective was offered. Some studies also included data from health care professionals (HCPs) which we did not include in our review [14, 17, 20, 22]. Given the small number of eligible studies, studies were also included if the participants were primarily people who had cancer (over 75% of participants) [18, 21]. We did not include the first order content in our analysis when the data pertained to a non-cancer patient. In total across all the included studies, there were 197 patient participants, of whom 87 were explicitly identified as patients not receiving end-of-life care. In so far as possible, data was only extracted for the patients who were people with cancer who were not receiving end-of-life care.

Reflecting the various end-of-life care models around the world, the services that were declined or utilised by patients varied across the studies. For example, end-of-life services were delivered at home [14], in a hospital-based hospice unit [20], or as home-based palliative care [22]. At times this was ambiguous because the authors were grouping hospice and palliative care together (as defined in the introduction) or did not describe the model of care in detail. For example, one included study from the UK described their model of care as “community-based palliative care services are delivered by hospices, which are specialist palliative care inpatient units, commonly with a team of clinical nurse specialists and doctors who will visit patients at home and help to co-ordinate care” [22]. The model of care seemed to be most influential in the US where the policy of stopping curative treatment.

### Factors involved in delaying end-of-life care

Our analysis was aided by collating the themes in each paper (see Table 3).

**Fig. 2:**
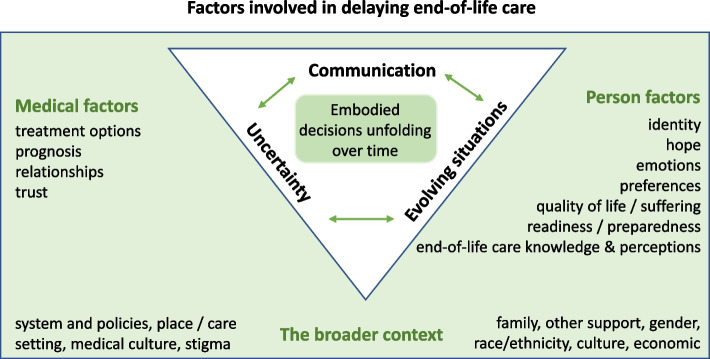
Factors involved in delaying end-of-life care

As shown in Fig. 2; Table 3, the included studies can be reconceptualised with the key concept (third order construct) of an embodied decision to accept or delay end-of-life care as unfolding over time. We synthesised the second order constructs into the three interpretive themes. Each interpretive theme was a primary influence on how that decision unfolded for patients were (1) the communication they received about end-of-life care; (2) uncertainty around their prognosis, and (3) the evolving situations in which the patient and family found themselves. These three interpretive themes were in turn shaped by a range of factors that we integrated into three sets, person factors, medical factors and contextual factors that helped to shape the decision-making process. We discuss the key concept, each of our three interpretive themes and sub-themes below, taking the broader factors from the literature into account throughout.

End-of-life care decision-making is a process influenced by the person, medical and contextual factors and interpretive themes, communication, uncertainty, and the evolving situations. For patients whose decision to decline end-of-life care never changes, the medical and contextual factors in the model are not sufficiently influential to move them over the threshold of opting for end-of-life care. In other words, there is too much uncertainty, insufficient communication about what end-of-life care can do for them, and the situation does not mean the person considers that care as a viable choice (at that point in time). Patients may have still been open to revising their decision if these change.

### Key concept: embodied decisions unfolding over time

Some studies, particularly the earlier ones, found that the decision to accept or decline end-of-life care was a binary decision while later studies identified how these decisions were much more responsive to situations and timings, such that end-of-life care decision-making changes and evolves over time. In their US study, Spencer et al. showed how patients’ decision-making was less a matter of declining end-of-life care than a postponing or delaying. They termed this a ‘soft no’ [21]. The recursive process of suffering, struggling and, for some, moving on to a settling phase aligns with the developmental transition from living with to dying from cancer [18, 19]. 

In reviewing these studies, we realised that focussing on declining end-of-life care framed decision-making as an event rather than a process. However, there were some patients in the studies who were identified as never accepting end-of-life care. For example, the four out of 26 participants in the Spencer study that were defined as a ‘hard no’ to end-of-life care [21]. 

This delaying notion of ‘embodied decisions unfolding over time’ emphasises the iterative, dynamic, situational, contextual and relational nature of decisions for end-of-life care that are grounded in people’s physical experiences. Decisions about when (and for some, whether at all) to accept end-of-life care are made in a complex system with preferences shifting over time, in relation to the embodied experience of life-limiting cancer [16, 19, 21]. 

### Interpretive theme: communication: timing, services, and content

The importance of communication was identified by all studies. More specifically, the timing, how services were described, and the content of the conversations about the transition to end-of-life care were crucial in decision-making. The notion of time is vital to end-of-life decision-making both in terms of when during the diagnosis and prognosis trajectories the conversations happen, and how frequently. Many participants in the studies said they would delay deciding until they felt the time was right [19, 21]. If the conversations occurred too early, then patients’ need for end-of-life care was sometimes not yet apparent to them and their family, and the referral was described as ‘distressing’ [22]. Patients wanted end-of-life care “when it’s going to be helpful”, which is to say they could not see the need or the benefit of this yet [22]. However, the need or potential to benefit was clearer to the HCPs making the referral and sometimes to families [14, 19, 22]. If the conversations occurred too late, then there was less time to benefit from the services [14]. 

As well as the timing of the communication, the way in which the communication occurred, and how end-of-life care was presented during this communication influenced decision-making [14, 15, 17–22]. Lin et al. found for some patients there was an absence of opportunities to choose palliative care (though this was reported by their HCP participants) [20]. When patients could see the alignment between their (perceived) needs and what was on offer, then the communication about end-of-life care further confirmed that they were or were not ready for it. This also related to patients not knowing or accepting they were dying [14, 22]. The importance of patients’ awareness of what end-of-life care is was identified in many of the studies [14–17, 20–22]. In some studies, patients discussed being confused about how end-of-life care fitted with the current care they were receiving (e.g., curative treatments, palliative care, home support services) [14, 18, 19, 21]. In the Vig et al. study, there were information and admissions visits and referrals by HCPs which added to patients’ and family members’ confusion as to what the appointment or referral was for [14]. 

The studies also concluded that the content conveyed during the conversation depended on the skill of the referrer [14, 15, 17, 18, 20, 22]. Communications about end-of-life care needed to be culturally appropriate as well as tailored to the person’s situation, language, and needs [15, 17, 18, 20, 22]. For example, Carrion and Frey et al. identified gaps in referrer and patient communication, and interpreted these as arising from a lack of understanding about end-of-life care as a concept, lack of shared cultural scripts, language issues, differences in religious beliefs, differences in cultural understandings of different perceptions of health and illness, and differences around caregiving expectations [15, 17]. Materials in other languages was recommended to facilitate the communication of end-of-life services, but these only addressed a small number of issues that were identified [15, 17]. 

### Interpretive theme: uncertainty

Several studies identified the uncertainty that patients experienced as they approached the end of life. This was related to the unknown length of time they had left to live as well as uncertainty about end-of-life care and how to access it [19, 22]. In some cases, the prognosis was concealed from the patient by the family and HCPs in an attempt to minimise their distress. However, in the Taiwan-based study by Lin et al., prognosis concealment served to provoke uncertainty about the future and provoke emotions in patients that were then covered up to protect family [20]. Some patients experienced prognostic uncertainty because both they and their clinicians were sceptical of the accuracy of the prognosis [22]. 

While uncertainty was primarily discussed by Spencer et al. and Waldrop et al., we consider it an important conceptual factor because it accommodates the need for contingency also opens up space for hope that further cancer treatment options could become available [19, 21]. Waldrop et al. described uncertainty as occurring over what the right thing to do was when there were no curative treatment options [19]. In some situations, the uncertainty resolved when HCPs were able to provide some certainty over the disease course, enabling patients to make the decision to access end-of-life care [21]. Spencer et al. concluded that the delaying decision-making approach is “a deeply longitudinal decision strategy in response to uncertainty” [21]. 

### Interpretive theme: evolving situations

Uncertainty as a concept depicted in Fig. 2 relates to the evolving nature of the person’s end-of-life trajectory. However, there were other aspects of patients’ situations that were changing and evolving. Four studies all acknowledged the dynamism of the situation as influencing end-of-life care decision-making [16, 18, 19, 21]. This was, in part, physical for the patient and how caregivers were coping [14, 16, 18, 19, 21]. Patients continued to reassess priorities and preferences based on their evolving situations, particularly around their quality of life, suffering and pain and how these needs were able to be met without end-of-life care [16, 18, 19, 21]. This points to the embodied aspects of decision-making, in that patients’ changing physical reality of living with cancer and its progression shaped the decisions they needed to make, including decisions about end-of-life care.

Spencer et al. theorised that patients who used a ‘soft no’ to the offer of end-of-life care meant that it left room in the future for a yes, depending how their (usually medical) circumstances evolved [21]. This clearly positions decisions as contingent and iterative (re)assessments of the current situations,highlighting that decision making is a process that unfolds over time. Waldrop et al. and Meeker et al. framed the decision to access end-of-life care as a major transition and turning point in the cancer treatment trajectory [18, 19]. For some patients, a developmental shift occurred physically, cognitively and in identity that facilitated them accepting end-of-life care [18]. Meeker et al. described how some patients moved from suffering and struggling phases towards a settling phase where they adjusted to a new phase of life and were aware of their limited prognosis [18]. These three interpretive themes, communication timing and content, uncertainty and evolving situations, are informed by person-specific factors, medical factors, as well as broader contextual factors to which we now turn.

### Influences: person factors

Individuals draw on their unique sense of self, preferences for care, embodied experiences of illness, attitudes towards dying, and knowledge and perceptions of end-of-life careto make decisions. Such decisions are also underpinned by emotions, including hope, and readiness for death. Four studies reported that the patients’ identity played a role in their decision-making [15, 17–19]. Identity was often conceptualised in terms of being in the non-dominant majority, such as being a migrant in a new land with a different culture and language, or having served as a veteran. Gender, ethnicity/race, and religion/spirituality as facets of identity were not well-explored in most studies. Studies also discussed changes in identity that occurred in terms of a developmental shift, as people moved towards accepting that they were dying [19]. 

Emotional aspects of decision-making were noted by eight of the nine studies [14–20, 22]. Patients in these studies discussed specific emotions such as fear (of death, of leaving negative memories), shame, a sense of loss and powerlessness, and the affective imperative to stay positive. Hope was another major facet that patients talked about as part of their decision-making – hope for more time and hope that during that time, further curative treatments would become available. As Waldrop et al. stated, “the equation of treatment with hope was a barrier to the consideration of hospice enrollment” [19]. 

Patients and their families’ preferences for care played a major role in seven studies. Some wanted to be cared for by their loved ones and thus chose not to access services. Pini et al. and Spencer et al. noted that patients preferred to remain with their current care team (oncology, palliative care) rather than transition to an unknown, new team [21, 22]. Meeker et al. was the only study to articulate that values (although not specified) guide preferences and therefore decisions for end-of-life care [18]. Preferences also informed patients’ views of what quality of life was acceptable to them and whether they needed further support to help them manage their physical symptoms. Notions of suffering and quality of life were identified across seven of the nine studies [14, 16, 18–22]. In the mixed-methods study Waldrop et al. measured quality of life (using the QLQ-30), which showed that non-end-of-life care patients had, at the time of measurement, a better quality of life, namely fewer compromising physical and functional changes, than patients receiving end-of-life care [19]. 

Patients’ physical state seemed to inform whether they felt they were ready for end-of-life care, i.e., their needs were not yet great enough to warrant it, and they would ‘know when’ the time was right [19, 21]. We put forward the term ‘embodied knowing’ to capture the sense, grounded in one’s body, that patients had of when the time was right to move towards end-of-life care. This form of ‘embodied knowing’ played a part in readiness, which was discussed as accepting or acknowledging dying, being ready to go, or a psychological and emotional preparedness for discussing end-of-life care [14, 16, 19, 22]. 

Some patients talked about how end-of-life care was only used when people are ready to die [15, 22]. This highlights the role of patients’ understanding and knowledge about end-of-life care. Seven of the studies identified patients’ knowledge of what end-of-life care is as important, with Frey et al. concluding that knowledge of end-of-life care is important for its utilisation [14–17, 20–22]. Some patients had had positive experiences and others negative experiences of end-of-life care, usually from people they knew using the service [15, 16]. Common beliefs included that end-of-life care was only appropriate for the last days of life, when there was nothing more to do, was for people who had given up, or who had no other support [14, 15, 17, 20]. Knowledge was often framed as a misunderstanding on the part of the patients rather than a failure of end-of-life providers or other referring HCPs to explain and communicate in a way that was comprehensible to patients and their families [14, 17, 22]. 

Together, the above factors comprise the ‘how’ individuals approach decision-making using their own worldview, emotions, preferences, embodied knowing, and beliefs about what is a ‘good’ death and what end-of-life services can do for them to decide whether and when to access delay or decline them.

### Influences: medical factors

The end of life is heavily influenced by the medical system, those who work in it and what treatments are available. The studies highlighted the key role of HCPs in patients’ decision-making, in particular, how they communicated with patients and their families about their situation, services and options. The availability of treatment options shaped whether patients viewed end-of-life care as the right choice, or whether it was time for patients to consider end-of-life care or delay the decision. This was identified in all studies. Decisions about end-of-life care included cultural considerations for those in minority groups, and most crucially whether end-of-life care was culturally acceptable as an option [15, 17, 20]. The major example of conflicting conclusions across the nine papers was whether palliative care supported or inhibited the transition to end-of-life care. Waldrop et al. and Pini et al. discussed palliative care as supporting conversion to end-of-life care whereas Spencer et al. concluded that the stabilising influence of palliative care meant that a treatment-focused paradigm could be maintained [19, 21, 22]. One possible interpretation of this discrepancy is that it depends on the healthcare contexts and HCPs’ communication.

The importance of patients understanding their prognosis as a facilitator of moving towards accepting end-of-life care was identified in six studies [14, 15, 18–20, 22]. Most patients wanted to know about their prognosis, but not all. Patients were fully reliant on HCPs to share this information with them in a manner that they could understand. Meeker and colleagues noted that patients in their study talked about wanting to receive their prognosis from a HCP with whom they had a prior relationship [18]. As some researchers noted, the concealment of prognosis is a cultural norm in some Asian cultures [17, 20]. This was framed as a lack of communication skills and honesty among medical staff and as impacting the HCP-patient relationship and trust in the Lin et al. study, where a patient described as a huge surprise when she found out how long she had left [20]. Having a relationship with HCP was important for some patients, who did not want to move care providers because it would disrupt the continuity of care they experienced [14]. Others felt the current care was meeting their needs and saw no reason to change providers [16, 21]. Some patients talked about wanting to be cared for by someone from the same cultural background and that the end-of-life care provider was not able to provide that [17]. This ties into notions of trust with and in HCPs. Patients highlighted trust as an important part of their relationship with providers. Six of the nine included studies discussed trust as a factor in end-of-life care decision-making [14, 16–18, 20, 21]. HCPs were viewed by some as trusted advisors who could help inform decision-making, especially when there was uncertainty [21]. Trust in providers was facilitated by clear and consistent information about care and one’s health status [18]. Some patients and families from ethnic minorities had previous experiences of discrimination, causing mistrust of health professionals and the health care system in general [17]. 

### Influences: the broader context

The context in which decisions about whether or when to access end-of-life care was discussed by eight articles [14–21]. We conceptualised this broader context as a continuum that at one end was more medically and system oriented, and at the other was more person-oriented (see Fig. 2). Taken together, the context bridges and situates the person within the medical setting that heavily influences decision-making.

#### Person-specific contextual factors

All studies emphasised the role and influence of family (including significant others) in decision-making at the end of life. If both patients and family were not ‘ready’ for end-of-life care, there were resultant delays or end-of-life care was declined altogether [14, 22]. Conceptualising spouses and family members together with the patient as a decision-making unit was particularly important in seven studies [14–18, 20, 21]. For many patients, the perceived benefits to others, over oneself, was a reason to keep pursuing active treatment and delay or decline end-of-life care [15, 20, 22]. A few participants mentioned they wanted to be a role model to one’s family of fighting cancer [18, 20]. Some people wanted to have family around them and so did not wish to leave home to an in-patient unit [16, 17]. Being able to have family comfortably accommodated when visiting in-patient units was important to some people in their decision-making process [16, 17]. 

Whether participants had family to care for them at home or not was a strong consideration in receiving formal care [15, 19, 22]. At the same time, some family members expressed concerns about their ability to provide care for the patient at home [14]. Waldrop et al. found that patients not receiving end-of-life care as compared to those who were reported lower levels of social support, as well as the need for support from relatives and friends [19]. For some patients, end-of-life care decision-making may be delayed because support networks were meeting their needs, including family members having become expert caregivers over many years [21]. Several studies reported that patients and families were unclear on what additional benefits end-of-life care offered [14, 20–22]. 

Several studies mentioned the role of culture in end-of-life care decision-making [15, 17, 20]. They identified the culturally specific concepts: modesty, shyness, personal dignity, shame, filial piety, and notions of not wanting to receive welfare as being relevant to cultural values and norms. Frey et al. explained the challenges to end-of-life care use as ‘a matter of culture’, noting the different “ways of doing and thinking” among the ethnic minorities they interviewed compared to New Zealand European culture [17]. Carrion focused on the cultural factors that contribute to the under-utilisation of end-of-life care by Latinos in the US [15]. Several participants said that unlike the US, they had no culture of end-of-life care, did not wish to admit they needed help, and found the health care workers’ visits an invasion of privacy. In other studies, there was less explicit attention to the role of culture as authors acknowledged the lack of diversity in the samples which predominately included White, middle-class participants [14, 16, 22]. Somewhat surprisingly, the influence or importance of gender in the decision-making process was not discussed or analysed in the studies beyond reporting the gender of participants. In one study gendered norms and assumptions about wives in caregiving roles were made [14]. 

Financial considerations came into end-of-life decision-making when patients and providers discussed the costs of care. The costs of care had several dimensions, such as the limited ability to pay for care, the cost of continuing active treatment, the hidden costs of being a caregiver, and the socio-economic disadvantage experienced in higher rates among ethnic minorities [14, 15, 17, 20]. In their comparison study, Waldrop et al. found that non-end-of-life care patients in the US had higher rates of financial problems compared to end-of-life care patients [19]. 

#### Medical contextual factors

Our analysis found that holistic, person-centred care are similarly defined across varied settings of delivery (i.e., end-of-life care at home vs. hospital vs. in-patient facility). However, these settings of care and what is available to patients differ across the included countries, and this likely influences patients’ views on end-of-life care. This analysis was hindered by the limited descriptions of the context or models of care for some studies. A range of health system and health policy factors were apparent as key parts of decision-making in the studies. Health system policies were described as forcing ‘terrible choices’ onto patients [21]. For example, patients had to choose between active treatments or end-of-life care [14, 15]. All of the studies that mentioned such barriers were based in the US where the health care system influenced end-of-life care decision-making through setting the cost of care [14, 15, 21]. Nevertheless, only two studies mentioned how place played a role in the decision-making process. Chapple et al. reported that desired place of death plays a role in decisions about where to receive care, while Frey et al. found that many patients viewed hospice as a place to die rather than a philosophy of care [16, 17]. Place also manifested in where some patients were given information. The non-end-of-life care Hispanic/Latino patients in the US all found out their terminal diagnosis from their primary physician whereas end-of-life care users learned this information during a hospital admission from an attending physician [15]. Place or care setting and the patient’s condition are connected. Conversations and end-of-life care decisions which take place in hospital are likely to differ from other contexts such as outpatient clinics and hospice buildings. Considering contextual factors such as the place and care setting where conversations that are expected to result in decisions or at least inform decision-making as well as the patient’s status appears to be important.

The relevance of the broader medical culture was also apparent in the studies’ findings. The culture of medicine to treat, even when it may have been futile, was particularly noted by Lin et al. as influencing end-of-life decision-making and also commented upon by Waldrop et al. and Meeker et al. [18–20] In the Lin et al. study, this attitude was further reinforced by a culture of defensive medicine where clinicians feared repercussions of being sued if they did not provide curative treatment [20]. Perhaps relating to the treatment imperative of medicine across many cultures, the studies demonstrated that stigma is attached to end-of-life care. This stigma was related to death and dying, and the type of service offered, namely where patients were expected to share things about themselves, talk about death and dying, and engage in activities [22]. In New Zealand, stigma and thus shame was attached to the end-of-life care which is free for patients and families, because it was assumed it was only for those who could not pay [17]. 

## Discussion

We begin this discussion by briefly returning to the review questions and considering them further based on the current findings and the wider literature. This systematic review and meta-ethnography sought to identify why patients with life-limiting cancer decline end-of-life care. However framing the question in this way assumed that patients *do* decline end-of-life care, whereas the findings demonstrate that many patients delay accepting end-of-life care rather than decline them altogether. The review also sought to identify what informs patients’ end-of-life care decision-making. This is captured with the synthesising model that shows how end-of-life care decisions are embodied and unfold over time, and identifies the main factors on both patients and families’ decision-making as involving three interpretive themes, namely uncertainty, communication, and the dynamic situation. Around these main themes, personal, medical, and contextual factors also influence in decision-making processes. We note that not everyone can access end-of-life care due to local availability, resourcing, eligibility policies, or the type of illness they have. For those who are referred, their experiences of end-of-life care decision-making (question three) is one that occurs over numerous occasions, often prompted by a change in situation (e.g., caregiving at home), change in embodied functioning and experience (e.g., functional status or quality of life) or a HCP referring them when curative treatments are no longer available. In terms of question four about the common characteristics of people who decline/delay end-of-life care, it appears minority ethnic groups face unique barriers around language, cultural fit of services, and trust with HCPs [15, 17]. However, trust was an issue that was identified across multiple studies with a range of participants, and has also been identified in other research on this topic [2, 14, 16–18, 20, 21, 25, 26]. 

Romo et al. also undertook a review on a similar topic [2]. Their article was located with our research strategy but was excluded because it was a review. Our findings, and particularly the model we have posited, has significant overlap but also some differences to Romo et al.’s findings [2]. Notably, our meta-ethnography did not identify decisional control as playing a key role, but it did identify uncertainty, emotions, and perceptions of end-of-life care which are not in Romo et al.’s findings [2]. Both reviews emphasise individual factors, communication, prognosis, the influence of others on decision-making, and the importance of the contextual environment. Though named differently, Romo et al.’s illness/health experience correspond to our preferences and quality of life / suffering factors [2]. 

Spencer et al., who used the Romo model as their framework, concluded that there was a need to move beyond cognitive- and emotion-based paradigms of patient education to recognise influence of the social context on end-of-life care decision-making [2, 21]. We concur that the context is crucial however, from our review, it is clear that for patients, the emotional aspect of decision-making must be acknowledged as well. We need a model that incorporates all of these elements and remains true to patients’ experiences and voices. We hope our model goes some way in doing this.

Our key concept of embodied decisions unfolding over time centres on the role of time in decision-making. Time was discussed in several studies, though it was not identified as a theme in any of the studies [15, 18, 19, 21, 22]. Part of Pini et al.’s engagement theme was understanding how end-of-life care could benefit present and future care [22]. Time intersected with communication, uncertainty and the evolving situation. Time meant uncertainty may resolve or persist in terms of how long someone has left.

Kaufman proposed the term reflexive longevity to refer to “an emergent form of life, a mode of knowledge, reasoning and embodiment that older persons and their families come to inhabit at the site where ethics, ageing, clinical technologies and life itself meet” [27]. Reflexive longevity shapes the way we live and medical decision-making, calculating the time left with respect to age, risk, death and likely success of medical interventions [27]. Reflexive longevity and the concept of time left are relevant to end-of-life care decision-making too. Time left is a discursive concept that shapes clinical and individuals’ decisions about what treatments to pursue or whether to opt for more palliative options. Patients may be ‘calculating’ time left when deciding to accept, delay or decline end-of-life care based on age, quality of life and suffering due to the effects of their illness and the nature of their anticipated dying. What has been communicated to them about their prognosis, treatment options and their evolving situation will also likely factor into their decision-making processes.

During our analysis, we observed that several factors that were not given sufficient weighting or were lacking altogether. Surprisingly, the studies undertaken did not discuss in much depth on the multiple and intersecting domains of health that inform decision-making, namely physical, mental, social, and existential/spiritual [28]. This may have been a study design issue where these factors were not explicitly enquired about or a reporting issue. Waldrop et al.’s mixed methods study added a unique finding, namely that patients who were accessing end-of-life care had worse symptom burden and a less manageable quality of life than non-end-of-life care patients [19]. In this study the physical and embodied nature of approaching the end of life was explicitly included and addressed in a way that was not seen in the other studies. Emotions/affect was an implicit factor in many papers. However, the explication of the emotional aspects of decision-making warrants further attention. In particular, the fear of dying seems pertinent. Similarly, existential or spiritual aspects of decision-making were only mentioned in passing by Waldrop et al. [19].

The embodied, lived experiences of illness (and life more generally) comprise an important part of the calculation or decision-making process [27]. Embodiment brings together temporal, cultural and biological aspects of life and the inevitable changes over the life course [29–31]. The issues and decisions people face as they approach the end of life are closely related to their embodied reality of decline and suffering, shaped by the material world around them [30]. One’s sense of self, time, daily life, and functional ability are disrupted by the bodily deterioratation associated with cancer [30]. For people with inoperable lung cancer, time is experienced as a clash between clock time, which the health system runs to, and embodied time, which is how individuals experience time existentially and as enmeshed in history, culture, space, relationships, and contexts [31]. ‘Embodied knowing’ alludes to the sense that patients had of when the time was right to move towards end-of-life care. The concept of a right time accords with a US study which found that 58% of family members of patients who died within a week of hospice enrolment considered that the referral was the right time [32]. On the other hand, as Pini et al. described, “Patients who had experienced palliative care almost unanimously wished it had happened earlier and the reality of being involved in these services was significantly different to their prior perceptions” [22]. Any conceptualisations of end-of-life care decision-making need to take account of embodied decision-making, because dying is always, already embodied.

Building on the notion of embodied knowing, one concept that we consider underemphasised which was explicitly questioned by Meeker et al. and Vig et al., is the assumption that end-of-life care and comfort care is the best focus for all people, regardless of the goals of care, cultural safety, religious or other mismatch between patients and their family and end-of-life care [14, 18]. Some study participants preferred to continue active treatment. The culture of medicine and society towards dying, in general, pervaded the studies. Treatment imperatives appear to be underpinning in some patients in the included studies’ decisions. For example, participants’ views were surmised as “treatment equals hope” or “treat or die”. The treatment imperative is difficult to overcome due to emotional, communicative and relational factors. Tate used conversational analysis to analyse doctor-cancer patient consultations, showing interactional hesitancy to discuss end-of-life options that superseded other policy and structural drivers for less medical intervention in end-of-life care [33]. Spencer et al. also noted the influences of physicians, the cultural authority of medicine and science, treatment structures and patients’ biographical histories as being relevant [21]. Several studies’ participants described how the precursor to considering end-of-life care was feeling or being told that nothing more can be done for them in terms of treatment. This affirms the unfounded notion that end-of-life care is positioned as ‘doing nothing’ in relation to curative treatment, despite active approaches to deal with not just physical, but social, spiritual and mental concerns. Borgstrom et al. go further to describe how non-intervention, or the more active form ‘not doing’, still needs to be done and note that passivity can be intentional [34]. 

The next step for research in this area is to consider whether this model coheres with what end-of-life care referrers think are the determining factors in patients’ and families’ decision-making processes. Further, it would be useful to explore what patients and families think, especially those who delay or decline end-of-life care and non-cancer patients. Embodiment, time, and the dimensions of health are all worthy of deeper exploration with people approaching the end of life. The model’s themes and factors can be used to address the barriers and reasons patients delay or decline end-of-life care (whilst accepting that end-of-life care is not the right choice for some people). Several articles offered strategies for addressing the reasons that patients or family members gave for not enrolling in end-of-life care or for overcoming identified barriers to enrolling. Vig et al. were the clearest in their framing of how providers can respond to specific concerns [14]. However, their suggestion to avoid the words ‘hospice’ or ‘dying’ when talking to patients was surprising to us, given the emphasis on acceptance of dying as part of the model of end-of-life care [35]. Another line of analysis to consider is whether the model reflects those who decide to accept end-of-life care. A quantitative review of the reasons people delay or decline end-of-life care will likely demonstrate other factors such as ethnicity/race and religion and other factors contributing to the inequities of access.

### Strengths and limitations

The meta-ethnography methodology was novel and enabled us to create a model of the studies, including a reconceptualisation of the data as embodied decisions unfolding over time. Team members’ varied backgrounds (sociology, health psychology, health promotion) and skills were helpful in focusing the topic and conceptualising the findings. This methodology also examines the contextual data about each study; however the variation of end-of-life care models was a factor that we were unable to explore in greater detail due to the lack of explanation of the operational model in some articles. Even though the studies were quite varied, from a number of different countries, cultures and health care systems, there remained a number of similarities in the decision-making processes around end-of-life care. This suggests that there may be some transferability of the proposed model and future research in various settings will be relevant to the field. In some studies that included both end-of-life care and non-end-of-life care patients, and cancer and non-cancer patients, it was sometimes unclear who was receiving end-of-life care and who was a non-cancer patient [16, 18–22]. This means we may have included data that was from patients who were receiving end-of-life care as well as non-cancer patients. As several of the included articles were included from our own records as opposed to the database searching, we cannot be confident we located all eligible studies.

## Conclusion

From this review we conclude that the decision to decline or delay end-of-life care is an ongoing responsive decision-making process that is personal, embodied, relational, influenced by the timing, content and presentation of services communication, shaped by uncertainty, and the patient’s evolving health situation. There are individual and medical contextual factors, including both individuals’ and medical cultures, that impact how and when decisions are made or postponed. We also conclude that time is central to patients’ end-of-life care decision-making, in particular, estimating how much time one has left and patients’ embodied knowing about when the right time for end-of-life care is. The increasing interest in the integration of hospice palliative care across the cancer care trajectory and earlier introduction of end-of-life care highlight the importance of these findings for improving access.

## Data Availability

All data (articles included in the review) is publicly available. Materials are included in the manuscript.
